# An evaluation of the N_2_O emission reduction potential of enhanced efficiency nitrogen fertilizers and application timing in a semi‐arid cereal cropping system

**DOI:** 10.1002/jeq2.70251

**Published:** 2026-09-07

**Authors:** Erin Daly, Cheyne Ogilvie, Reynald L. Lemke, Richard E. Farrell

**Affiliations:** ^1^ School of Environmental Sciences University of Guelph Guelph Ontario Canada; ^2^ Department of Soil Science University of Saskatchewan Saskatoon Saskatchewan Canada; ^3^ AAFC‐Saskatoon Research and Development Centre Saskatoon Saskatchewan Canada

## Abstract

Efforts to cut fertilizer‐related nitrous oxide (N_2_O) emissions require sustainable nitrogen (N) management that maintains crop productivity while reducing emissions. Technologies such as enhanced efficiency N fertilizers (EENFs) have potential to support best management practices by improving N use efficiency and lowering N_2_O emissions. This study (i) evaluated the effects of spring versus fall application of conventional urea and EENFs, including a polymer‐coated urea (PCU; ESN), a nitrification inhibitor (NI; eNtrench), a dual urease inhibitor (Limus), and a urease and nitrification inhibitor (UNI; SuperU), on N_2_O emissions from spring wheat over two growing seasons in central Saskatchewan and (ii) compared soil nutrient supply rates among conventional urea and the EENFs to determine whether soil N dynamics reflected their differing modes of action. Results indicate that significant N_2_O emissions reductions can be achieved in rainfed cropping systems by using a urea‐based EENF without crop yield penalties. In general, the greatest emissions reductions were achieved using EENF products that employ an NI; both the NI and the UNI reduced annual cumulative N_2_O emissions relative to urea by 8%–54% and 36%–64%, respectively (*p* < 0.05). Conversely, emissions from the PCU trended higher by 17%–41% relative to conventional urea, except when spring‐applied in Year 1, when PCU reduced N_2_O emissions relative to urea. Across all treatments, spring N fertilizer applications led to the greatest N_2_O emissions reductions with EENFs compared to fall N fertilizer applications. Overall, NI‐based EENFs show potential for reducing N_2_O emissions while maintaining wheat yields in Saskatchewan.

## Plain Language Summary

This study evaluated the ability of enhanced efficiency nitrogen fertilizers (EENFs) and the timing of their application to reduce nitrous oxide (N_2_O) emissions, a potent greenhouse gas (GHG), from wheat crops in a semi‐arid region. Several different types of EENFs were assessed, including nitrification inhibitors (NIs), urease inhibitors (UI), and polymer‐coated urea (PCU), applied either in the fall or spring over two growing seasons. Fertilizers containing NIs significantly reduced N_2_O emissions by up to 64% compared to conventional urea, and spring applications of EENFs generally led to greater reductions in emissions than fall applications. The PCU was less effective, sometimes resulting in higher emissions than urea. Critically, use of EENFs did not impact wheat grain yields, suggesting that NI‐based EENFs, especially when applied in spring, can effectively reduce GHG emissions from cereal cropping systems without sacrificing crop productivity.

**Abbreviations**
ANOVA: analysis of variance
DUI: dual urease inhibitor
EENF: enhanced efficiency nitrogen fertilizer
NI: nitrification inhibitor
PRS probe: Plant Root Simulator probe
UNI: urease and nitrification inhibitor

## 1 INTRODUCTION

Nitrogen (N) fertilizers are extensively applied across the Canadian Prairies to maximize the productivity of conventional field cropping systems (Awada et al., 2021; Statistics Canada, 2025). While synthetic N sources enhance crop yields and farm profitability, their use can also lead to substantial environmental consequences due to nutrient losses from the system (Ferland et al., 2024; Zhang et al., 2021). One such loss occurs in the form of nitrous oxide (N_2_O), a greenhouse gas (GHG) 273 times more potent than carbon dioxide (CO_2_) on a mass basis that also contributes to ozone depletion (Forster et al., 2021; Ravishankara et al., 2009). In Canada, the primary source of N_2_O emissions is the application of N fertilizers to agricultural soils. As of 2022, Canada's agricultural sector accounted for 76% of national N_2_O emissions (ECCC, 2024).

In response, the Government of Canada introduced an emissions reduction target for fertilizer‐related N_2_O emissions of 30% below 2020 levels by 2030 (Fertilizer Canada, 2022). In addition, there is a growing number of voluntary corporate scope 3 emission reporting and reduction initiatives that are bringing attention to nutrient management in Canada (Wilton et al., 2024). To meet this goal, sustainable N fertilizer management strategies that support environmental sustainability while maintaining crop yields must be identified, promoted, and adopted. One such strategy proposed is the 4R Nutrient Management framework of using the “right rate,” “right place,” “right time,” and “right source.” Development and verification of technologies that support 4R Nutrient Management are critical to improve the efficiency of N fertilizer use in Canada and across the globe. Among these technologies, enhanced efficiency N fertilizers (EENFs) offer a promising solution.

At a mechanistic level, EENFs function by (i) delaying the conversion of urea to ammonium (NH_4_^+^) or the nitrification of NH_4_^+^ to nitrate (NO_3_^−^) using enzyme inhibitors or by (ii) slowing nutrient release through a physical barrier, such as a polymer or sulfur coating, to better align N availability with crop uptake (Dimkpa et al., 2020; Halvorson et al., 2014). Depending on the specific formulation, EENFs may be integrated into conventional fertilizers or applied as stand‐alone products. While EENFs primarily align with the “right source” category of the 4R Nutrient Management framework, they can also impact the “right rate” by reducing overall N fertilizer requirements (Ferland et al., 2024; Tenuta et al., 2023), the “right place” by enabling safe seed placement with slow‐release formulations (DeBruin et al., 2021), and the “right time” by potentially mitigating the greater losses often associated with fall N fertilizer applications (Thilakarathna et al., 2020; Wood et al., 2024).

The decision to apply N fertilizer in the fall rather than in the spring is influenced by various factors (Thilakarathna et al., 2020; Wood et al., 2024). In temperate regions such as the Canadian Prairies, fall fertilization is often practiced due to its logistical benefits, including reduced spring workload and lower N fertilizer costs (Thilakarathna et al., 2020). However, the timing of N application can greatly affect the magnitude or susceptibility to loss of the applied N, particularly as N_2_O. Fall‐applied N can undergo nitrification in warm, moist soils before freezing temperatures set in, leading to NO_3_^−^ accumulation (Abalos et al., 2016; Lin et al., 2017; Wagner‐Riddle & Thurtell, 1998). Fertilizer products designed to slow N transformations in soil may help mitigate N losses during the spring thaw by limiting NO_3_^−^ accumulation and mitigating the subsequent release of N_2_O emissions during the nongrowing season (Ferland et al., 2024; Wagner‐Riddle et al., 2024).

Overall, while EENFs have the potential to mitigate N_2_O emissions, their effectiveness is largely influenced by environmental factors and other management practices. For example, Thilakarathna et al. (2020) observed that EENFs did not consistently reduce N_2_O emissions over two growing seasons from fall‐applied N fertilizer to spring wheat crops in Alberta, likely due to high baseline soil fertility and overarching influence of major precipitation events. Similarly, a field study in Saskatchewan spanning two growing seasons found that drought conditions masked the potential of EENFs to lower N_2_O emissions from canola (Phan, 2022). At the same time, EENFs have also demonstrated considerable potential for emission reductions. Wood et al. (2024) reported that two urea‐based EENFs, eNtrench and SuperU, decreased N_2_O emissions by up to 64% and 57%, respectively, from spring wheat crops in Manitoba. Similarly, Ferland et al. (2024) attributed substantially reduced N_2_O emissions over 3 years in Saskatchewan to improved N management, which included the use of an EENF over conventional urea, albeit at a comparatively reduced application rate.

Additionally, it is important to assess the effects on crop yields, as this will determine farmers' willingness to adopt an EENF product due to their greater cost compared to conventional fertilizers (Abalos et al., 2016; Kanter et al., 2015). It remains uncertain whether different EENF applications can mitigate N losses while maintaining or enhancing crop yield productivity in Western Canada. Indeed, many studies present conflicting yield effects from EENF use with no benefit (Fast et al., 2023; Thilakarathna et al., 2020; Woodley et al., 2020), increased yields (Drury et al., 2017; Fast et al., 2023), or reduced yields (Lin et al., 2017). The underlying causes of this variability are not yet fully understood but are likely due to the complex interactions among soil properties, crop characteristics, climate conditions, and management practices, all of which influence EENF effectiveness and contribute to their inconsistent performance across agroecosystems (Fan et al., 2022; Verburg et al., 2022).

Thus, further research across Western Canada is necessary to develop a robust understanding that will clarify these conflicting results. The objective of this study was to assess the impact of 4R management, specifically “right source” and “right time” principles on N_2_O emissions within a rainfed cereal production system in the Dark Brown soil zone of Saskatchewan. Overall, it aimed to (i) determine the N_2_O reduction potential of spring versus fall applications of conventional N fertilizers and EENFs, (ii) compare the nutrient supply rates of conventional fertilizers and EENFs, and (iii) determine the potential for various EENFs to mitigate N_2_O emissions relative to conventional N fertilizers.

## 2 MATERIALS AND METHODS

### 2.1 Site characteristics and experimental design

The field study was conducted over 2.5 years (i.e., Fall 2015 through Spring 2018) near Broderick, Saskatchewan. Soils were Orthic Dark Brown Chernozems with a clay‐loam texture. Plots (ca. 3 m wide × 10 m long) were established in the fall of 2015, then moved to an adjacent quarter section in the fall of 2016 for Year 2 (Figure S1). For both years, plots were established on canola stubble and seeded to Hard Red Spring Wheat. Plots were arranged as a randomized complete block design consisting of four blocks and eleven treatments per block. Treatment combinations consisted of two experimental factors: fertilizer source and application timing. As a result of the different application timings, the measurement year (henceforth referred to as the N year) was structured so that fall‐applied fertilizer was assessed from Fall 2015 to Fall 2016 (Year 1F) and from Fall 2016 to Fall 2017 (Year 2F). Similarly, spring‐applied fertilizer was evaluated from Spring 2016 to Spring 2017 (Year 1S) and from Spring 2017 to Spring 2018 (Year 2S) (Figure S2). The fertilizer sources and application timings investigated in this study are summarized in Table 1; management activities including fertilizer, seeding, and agrochemical application dates are summarized in Table S1.

**TABLE 1:** Fertilizer sources and application timings investigated in this study.

N source	ID	Manufacturer	Mode of action	Active ingredient	Application timing
ESN	PCU	Nutrien	Controlled release	Polymer coating	Spring and Fall
SuperU	UNI	Koch Agronomic Services	Urease and nitrification inhibition	*N*‐(butyl) thiophosphoric acid triamide (NBPT) and dicyandiamide (DCD)	Spring and Fall
eNtrench	NI	Corteva Agriscience	Nitrification inhibition	Nitrapyrin	Spring and Fall
Limus	DUI	BASF	Dual‐action urease inhibition	NBPT and *N*‐(propyl) thiophosphoric triamide (NPPT)	Spring and Fall
Urea	Urea	–	–	–	Spring and Fall
Check	Check	–	–	–	–

Abbreviations: DUI, dual urease inhibitor; NI, nitrification inhibitor; PCU, polymer‐coated urea; UNI, urease and nitrification inhibitor.

### 2.2 Static chamber emission data collection and processing

Custom nonsteady state vented chambers constructed using polymethyl methacrylate measuring 22 cm × 45.5 cm × 15 cm (W × L × H) were installed parallel to crop rows (25 cm spacing), with two chambers placed in each plot (one on‐band and one off‐band) and remained in place for the entire year except when removed for seeding and tillage operations. Because fertilizer was mid‐row banded between every second crop row, area‐based cumulative N_2_O emissions were calculated by averaging fluxes from the on‐ and off‐band chamber in each plot. This approach mitigated overestimation of field‐scale emissions if only on‐band measurements were used. Emissions of N_2_O were measured 1x–2x weekly throughout the duration of the growing season, with sampling intensity increasing up to 3x weekly during the spring thaw period and after seeding and fertilization operations. Samples were also collected within 24 h after a major precipitation event. Gas samples were collected between the hours of 10:00 a.m. and 2:00 p.m.

Gas samples were collected from each chamber on an 18‐, 36‐, and 54‐min time step. Three sets of paired ambient air samples were collected as time zero gas concentrations and for calculation of the minimum detectable concentration difference, as per Yates et al. (2006). Samples were collected using 20‐mL syringes fitted with a 22‐gauge needle and injected into evacuated 12‐mL Exetainer vials and stored at 4°C until analysis via gas chromatography using a Bruker 450 GC (Bruker Biosciences Corporation).

Daily fluxes were estimated by fitting linear or exponential regression equations to the concentration versus time data using the Hutchinson Mosier Regression (HMR) model (Pedersen et al., 2010), a modified Hutchinson–Mosier method (Hutchinson & Mosier, 1981) in R (R Development Core Team, 2011). Cumulative fluxes were calculated using an area‐under‐the‐curve analysis of the daily flux versus time curves and reported as the average of four reps (Amadi et al., 2016; David et al., 2018; Engel et al., 2010). Cumulative fluxes for fall and spring N applications for year one included flux measurements from October 26, 2015 to November 16, 2016 (Year 1F), and from May 9, 2016 to April 27, 2017 (Year 1S), respectively. For Year 2, cumulative fluxes were calculated from October 31, 2016 to October 13, 2017 for the fall N application (Year 2F), and from May 15, 2017 to May 4, 2018 for the spring N application (Year 2S).

Fertilizer‐induced emissions (FIEs; kg N_2_O‐N ha^−1^) were defined as the amount of N_2_O emitted as a result of the N fertilizer application and were calculated using Equation (1):

(1) $$\mathrm{FIE}=E_{N}-E_{0}$$

where *E_N_* and *E_0_* are total annual N_2_O emissions (kg N_2_O‐N ha^−1^) associated with a given N source and the 0N check plots, respectively.

The emission factor (EF) for an N source was defined as the percentage of applied N emitted as N_2_O, calculated using Equation (2):

(2) $$\mathrm{EF}=\frac{\mathrm{FIE}}{N_{a}}\times 100$$

where *N_a_* is the fertilizer rate (kg N ha^−1^).

The N_2_O emissions intensity (EI) was defined as the amount of N_2_O emitted per unit of output (i.e., grain yield) and was calculated using Equation (3):

(3) $$\mathrm{EI}=\frac{\sum E_{N2O}}{Y_{t}}$$

where Σ*E*_N2O_ is the annual cumulative N_2_O (kg N_2_O‐N ha^−1^) emission and *Y_t_* is the grain yield (Mg grain ha^−1^) associated with a given treatment.

### 2.3 Soil and plant sample collection and processing

Soil samples were collected from each plot at depths of 0–15 cm and 15–30 cm using a probe (2.5 cm i.d.) on‐ and off‐band. Soil samples from the fall‐applied N treatments were collected 2 weeks after the fall fertilizer application and following snowmelt after spring thaw. All treatments (fall‐ and spring‐applied) were sampled 2, 4, and 6 weeks after spring seeding and fertilizer application. Samples were analyzed for plant available N (NO_3_^−^ and NH_4_^+^) by extracting 5 g of air‐dried soil with 50 mL of 2 M KCl and analyzed with a Segmented Flow Autoanalyzer (Technicon) following the protocol of Kalra et al. (2007).

Square meter plant samples were collected prior to harvest from each plot. Total biomass and seed yield were recorded and used to calculate harvest index. Subsamples of grain and straw were collected and ground to measure carbon (C) and N content using dry combustion in a Leco TruMac CNS Analyzer (Leco Corporation). In Year 2, a severe hailstorm resulted in the loss of harvestable crop at the field site. Estimated total biomass yields were collected using a forage harvester to collect the remaining biomass from the center six rows of each plot on July 27, 2017.

### 2.4 Plant Root Simulator probe installation and processing

Plant Root Simulator (PRS) probes (Western Ag Innovations) were used to measure NO_3_^−^ and NH_4_^+^ release by installing six probes (three anion and three cation) within fertilizer bands in each plot. PRS probes are electrostatically charged resin membranes that can be positively charged (i.e., cation resins for NH_4_^+^) or negatively charged (anion resins for NO_3_^−^). PRS probes were selected because they integrate N supply over time while providing a less labor‐intensive approach for assessing whether soil N dynamics reflected the differing modes of action of the EENFs (Harrison & Maynard, 2014; Owens et al., 2019).

Unlike conventional soil inorganic N extractions, which provide a snapshot of inorganic N concentration at the time of sampling, ion exchange membranes like PRS probes integrate soil inorganic N supply over a deployment period. Therefore, this method accounts for the dynamic nature of soil N cycling and provides a measure of cumulative N availability, which can be considered more representative of the soil N supply to crops (Harrison & Maynard, 2014). In Year 1, probes in spring‐applied treatments were exchanged every three days over a 30‐day period from May to June 2016, while probes in fall‐applied treatments were exchanged every five days beginning October 2016, including an overwinter period from November 2016 to March 2017. During each exchange, new probes were installed in the same location, while the retrieved probes were cleaned and sent to Western Ag Innovations for analysis.

Probes were exchanged frequently as prolonged deployment can result in resin saturation, which is a potential limitation of this method. Of note, the outputs of PRS probes are not directly comparable to traditional soil extractions. While soil extractions quantify inorganic N concentrations (mg kg^−1^), PRS probes measure the cumulative supply of ions to the resin over the burial period (mg cm^−2^ burial^−1^) (Harrison & Maynard, 2014; Qian & Schoenau, 2002). Due to funding constraints, NO_3_^−^ and NH_4_^+^ release from the fertilizer bands was monitored via PRS probes in only one year (fall and spring).

### 2.5 Statistical analysis

Preliminary data analyses included tests for normality and homogeneity of variance using the D'Agostino–Pearson and Bartlett tests, respectively. Data that were not normally distributed were log_10_ transformed prior to analysis and back‐transformed in tables and graphs for presentation. Treatment effects were determined by three‐way analysis of variance (ANOVA) with application timing, N‐year, and treatment (N product) as the main‐effect factors. Because application timing was only applicable to fertilized treatments, the unfertilized control was excluded from the initial three‐way ANOVA. Three‐way interactions generally were not significant (*p *> 0.05), though significant two‐way interactions (generally involving application timing or N‐year) were common. To facilitate comparisons among all treatments, including the unfertilized control, the data were subsequently stratified by N‐year and application timing, and one‐way ANOVAs were performed within each N‐year × timing combination, with treatment comparisons made using the least significant difference test. All statistical analyses were performed using CoStat software (version 6.451, CoHort software).

## 3 RESULTS

### 3.1 Site weather

**FIGURE 1:**
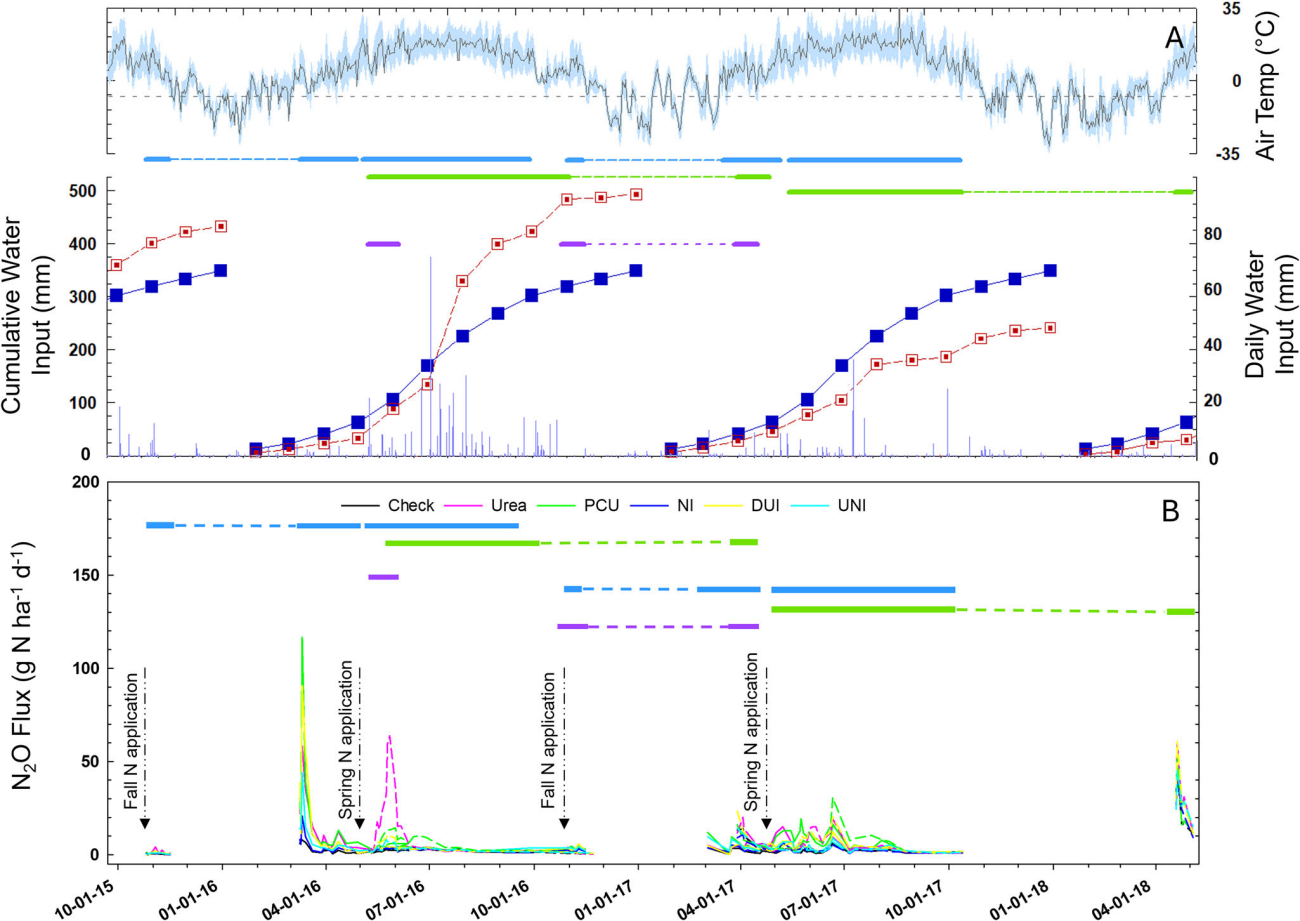
Climate and weather data summary (A) and daily N_2_O fluxes measured from plots receiving fall (solid lines) and spring (dashed lines) fertilizer applications (B) for the duration of the study (October 2015–May 2018). In panel A, precipitation is reported as cumulative (hollow red square) rainfall during the study period, together with the long‐term (20 years) average precipitation (shaded blue square). Relative daily precipitation is shown as light blue bars. The range in daily air temperature is shown as the blue shaded area, with the average daily temperature plotted as the solid gray line inside the shaded area. In both panels, the blue and green horizontal lines delineate the periods during which greenhouse gas (GHG) samples were collected following a fall N application or a spring N application. The short horizontal purple lines delineate the periods during which the Plant Root Simulator (PRS) probes were deployed to monitor nitrogen supply rates (NSR). The dashed lines mark the overwinter periods during which GHG and NSR samples were not collected. DUI, dual urease inhibitor; NI, nitrification inhibitor; PCU, polymer‐coated urea; UNI, urease and nitrification inhibitor.

Average annual precipitation for the site is 348 mm (30‐year normal), of which 70% occurs from May to September (Environment Canada, 2022). In 2015 and 2016, total annual precipitation exceeded the 30‐year normal by 24% and 41%, respectively, but was 31% below average in 2017. Growing season precipitation followed a similar trend. Fall precipitation between harvest and soil freezing was greater (64%–105%) for all 3 years of this study compared to the average, whereas spring precipitation from soil thaw to seeding was reduced (20%–51%) (Figure 1).

### 3.2 Ammonium and nitrate supply rates from PRS probes

**FIGURE 2:**
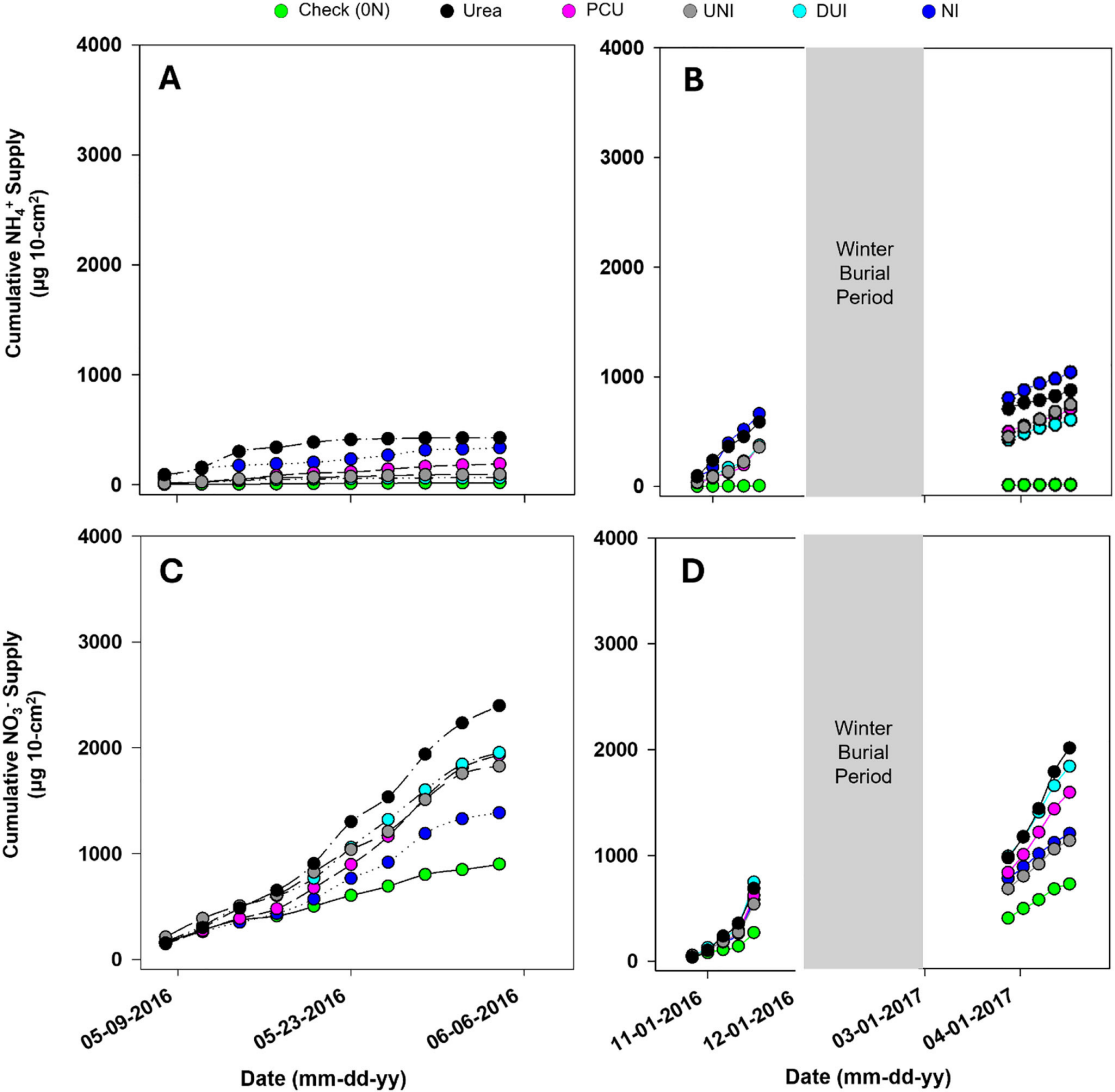
Cumulative supply rates of ammonium (NH_4_^+^) and nitrate (NO_3_^−^) from nitrogen (N) fertilizer treatments applied in spring (panels A and C) or fall (panels B and D) 2016. Supply rates were determined using Plant Root Simulator (PRS) probes that were exchanged every third day during the spring deployment and every fifth day during the fall deployment. (1) The set of probes installed on November 16, 2016 in the fall fertilized treatments remained in the soil overwinter and were recovered on March 28, 2017 and (2) the axes differ between panels. DUI, dual urease inhibitor; NI, nitrification inhibitor; PCU, polymer‐coated urea; UNI, urease and nitrification inhibitor.

Regardless of N fertilizer application timing, NH_4_^+^ supply rates were lowest in the check (0N) plots (i.e., <2.0 µg NH_4_^+^ 10 cm^−2^ 3 day^−1^ following spring application and <4.0 µg NH_4_^+^ 10 cm^−2^ 3 day^−1^ following fall application) (Figure 2A,B). The PRS probes left to overwinter following the fall fertilizer application showed minimal change in the NH_4_^+^ supply rate in the check plots over spring thaw (Figure 2B). Conversely, NH_4_^+^ data from the overwintered probes suggest that NH_4_^+^ was released from the fall‐fertilized treatments over the winter (Figure 2B).

Further, NH_4_^+^ supply rate and cumulative NH_4_^+^ supply were greatest for the urea and the nitrification inhibitor (NI), and lowest for the polymer‐coated urea (PCU), urease and nitrification inhibitor (UNI), and dual urease inhibitor (DUI) (Figure 2A,B). Moreover, the DUI yielded supply rates and cumulative NH_4_^+^ supplies that did not differ from the check plots when spring‐applied. In general, NH_4_^+^ supply rates and cumulative NH_4_^+^ were significantly (*p *< 0.001) lower following all spring N applications compared to when N fertilizer was fall‐applied (Figure 2A,B).

Initial NO_3_^−^ supply rates in the check plots were approximately 10x higher than NH_4_^+^ supply rates and were greater at the start of the spring sampling compared to the fall (Figure 2C,D). Additionally, while NH_4_^+^ supply exhibited only a slight increase during the 30‐ to 45‐day period following N application, NO_3_^−^ supply in the check plots increased at both sites across both N application timings. Regardless of fertilizer source, NO_3_^−^ release remained relatively slow for the first 12–15 days following spring fertilization, with PCU and the NI exhibiting the lowest rates. After this, NO_3_^−^ release rates increased for all fertilizer types, with the NI maintaining the lowest rate, resulting in the lowest cumulative NO_3_^−^ release (Figure 2C). Following fall N application, NO_3_^−^ release rates remained low, with no significant differences observed among treatments during the fall monitoring period. However, there was a substantial increase in NO_3_^−^ supply rates across all plots during the spring thaw (Figure 2D).

Regardless of application timing, PCU delayed NH_4_^+^ production in the soil. However, once released, NH_4_^+^ was rapidly converted into NO_3_^−^. Similarly, stabilized EENF products containing a urease inhibitor (both the DUI and UNI) slowed NH_4_^+^ release, but the released NH_4_^+^ was quickly transformed into NO_3_^−^. In contrast, EENF products with an NI had minimal impact on NH_4_^+^ release or production. However, the NI effectively slowed the conversion of NH_4_^+^ to NO_3_^−^. In general, the UNI maintained lower NH_4_^+^ supply rates and slowed the conversion of NH_4_^+^ to NO_3_^−^ relative to urea (*p* < 0.05).

### 3.3 Daily N_2_O emissions

#### 3.3.1 Year 1 (Fall 2015–Spring 2017)

In the first growing season, fall N application did not induce a large episode of N_2_O flux before soil freeze‐up, irrespective of N source. In general, daily N_2_O fluxes were low, though relatively large fluxes (>50 g N_2_O‐N ha^−1^) were observed during the spring thaw following the fall N application and post‐spring N application (Figure 1B). Spring thaw fluxes peaked on March 11, 2016, with the greatest fluxes originating from the plots receiving fall applications of PCU (*x̅* = 117 ± 76 g N_2_O‐N ha^−1^) and the lowest from the check plots (*x̅* = 8 ± 5 g N_2_O‐N ha^−1^). Within two weeks, elevated emissions associated with the spring thaw had returned to baseline values for all treatments.

The flux peak captured following spring N application was observed over a three week period beginning on May 27, 2016, with peak fluxes recorded from the urea plots (*x̅* = 64 ± 46 g N_2_O‐N ha^−1^). Fluxes from the EENF products were generally lower and not greater than baseline values, except for the PCU, which had a smaller, but longer period of heightened emissions compared to urea, peaking on June 1, 2016 (*x̅* = 14 ± 7 g N_2_O‐N ha^−1^), four weeks after spring N application (Figure 1B).

#### 3.3.2 Year 2 (Fall 2016–Spring 2018)

Spring thaw fluxes associated with the Fall 2016 N application were low (𝑥̅ = 6.2 ± 3.8 g N_2_O‐N ha^−1^ day^−1^). However, early spring had several small freeze‐thaw events (each lasting four to six days) prior to the final thaw event in mid‐March. Because of their short duration and unpredictability, the N_2_O emissions during these thaw events were not captured. Consequently, it is likely that some N_2_O emission events in Spring 2017 were missed (Figure 1B).

The flux peak associated with spring fertilizer application was smaller than the year prior, peaking four days after N fertilizer application on May 15, 2017. The treatment emission trend observed on this day was: Urea > PCU > DUI > UNI > NI > Check. Like 2016, fluxes from the PCU remained elevated for a longer period than those for the other treatments, with peaks on May 26, 2017 (𝑥̅ = 19.2 ± 13.5 g N_2_O‐N ha^−1^ day^−1^) and May 31, 2017 (𝑥̅ = 12.1 ± 8.9 g N_2_O‐N ha^−1^ day^−1^) (Figure 1B).

### 3.4 Annual cumulative N_2_O emissions, FIEs, yield‐scaled emissions, and EFs

Annual cumulative N_2_O emissions, FIEs, yield‐scaled emissions (YSEs) and EFs are summarized in Table 1. ANOVA for annual cumulative N_2_O emissions revealed that all three main effect variables (i.e., application timing, N‐product, and N‐year) had an impact on emissions (*p *= 0.06, *p *< 0.001, and *p *< 0.01, respectively). Whereas both two‐way and three‐way interactions involving application timing and N‐year were significant (*p *< 0.001 and *p *< 0.05, respectively), two‐way interactions involving N‐product and either application timing or N‐year were not significant (Table S2).

**FIGURE 3:**
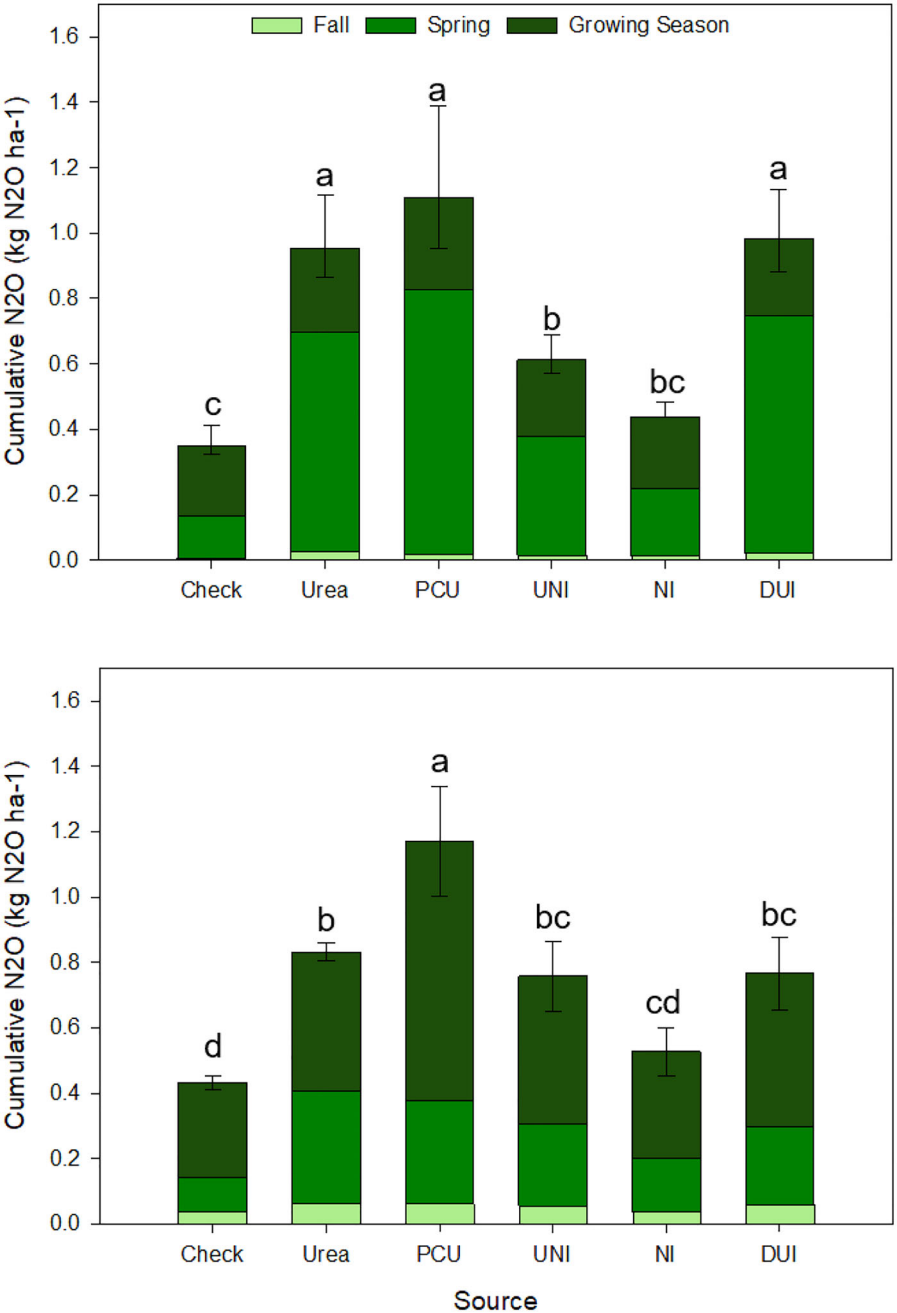
Cumulative N_2_O emissions associated with fall application of the conventional N fertilizer (urea) and enhanced efficiency N fertilizers (polymer‐coated urea [PCU], urease and nitrification inhibitor [UNI], nitrification inhibitor [NI], and dual urease inhibitor [DUI]) for Year 1F (2015–2016; top panel) and Year 2F (2016–2017; bottom panel). Error bars represent the standard error of the mean; within years, bars labeled with the same letter are not significantly different (least significant difference [LSD], *p* = 0.05).

**FIGURE 4:**
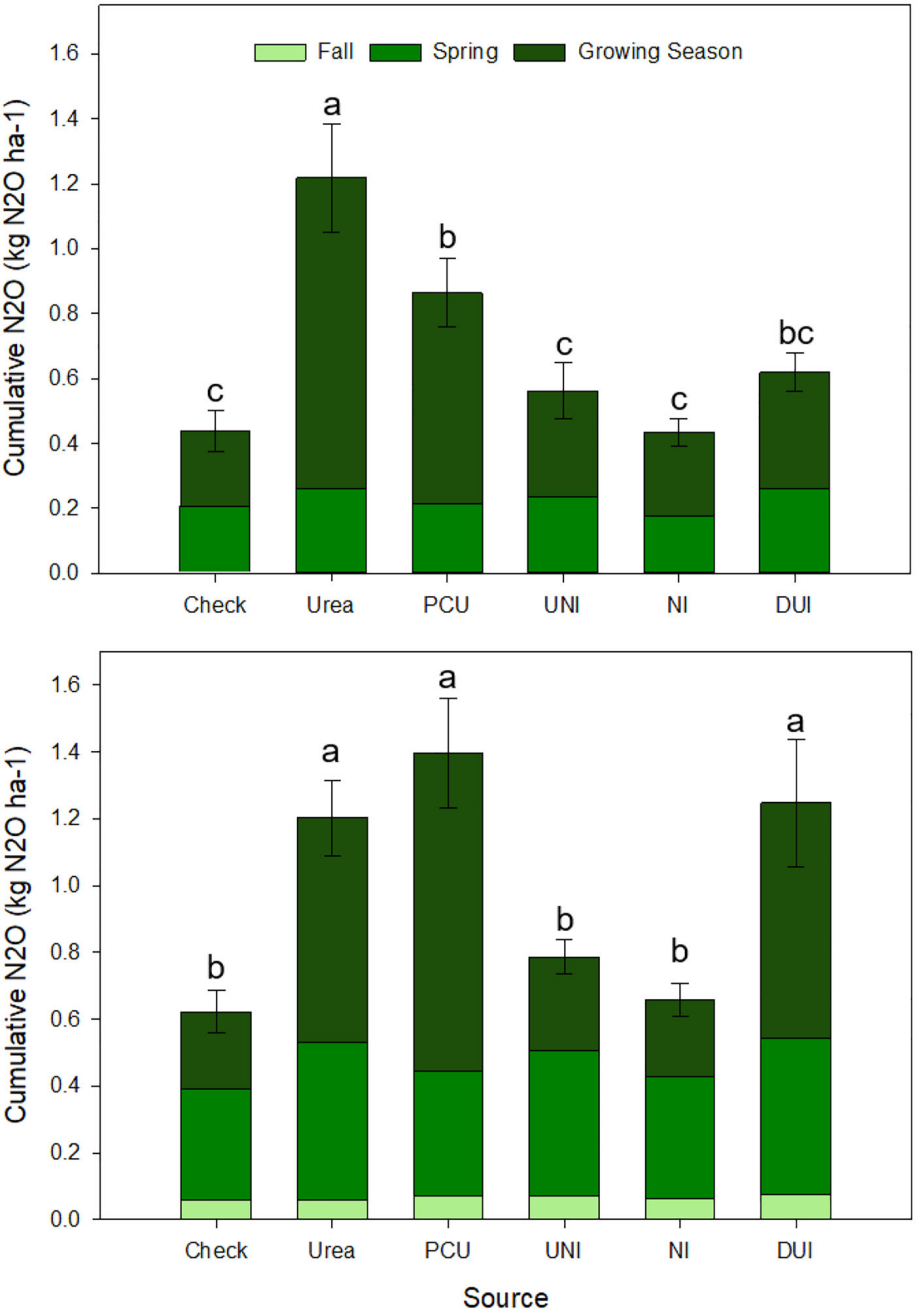
Cumulative N_2_O emissions associated with spring application of the conventional N fertilizer (urea) and enhanced efficiency N fertilizers (polymer‐coated urea [PCU], urease and nitrification inhibitor [UNI], nitrification inhibitor [NI], and dual urease inhibitor [DUI]) for Year 1S (2016–2017; top panel) and Year 2F (2017–2018; bottom panel). Error bars represent the standard error of the mean; within years, bars labeled with the same letter are not significantly different (least significant difference [LSD], *p* = 0.05).

The check plots maintained the lowest annual cumulative emissions, ranging from 0.37 to 0.62 kg N_2_O‐N ha^−1^ (Figures 3 and 4). Regardless of timing or year, emissions from the PCU trended higher by 17%–41% relative to conventional urea, except when spring‐applied in Year 1, when ESN reduced N_2_O emissions relative to urea, but not to the extent of other EENF products. Conversely, urea‐based EENFs containing an NI (i.e., the NI and UNI) consistently reduced annual cumulative N_2_O emissions relative to urea by 8%–54% and 36%–64%, respectively (*p* < 0.05). Limus inconsistently reduced N_2_O emissions relative to urea in 50% of cases; otherwise, emissions did not differ (Table 2; Figures 3 and 4).

**TABLE 2:** Fertilizer‐induced N_2_O emissions (FIEs), emission factors (EFs), and yield‐scaled emissions (YSEs) following a fall or spring fertilizer N application.

N source	EENF mode of action	Cumulative N_2_O^a^ (kg N ha^−1^)	FIE^b^	EF^b^	Yield‐scaled N_2_O^b^ (kg N Mg^−1^ grain)	Cumulative N_2_O^a^ (kg N ha^−1^)	FIE^b^	EF^b^	Yield‐scaled N_2_O^b^ (kg N Mg^−1^ grain)
		**Fall applied**							
		**2015–2016**				**2016–2017**			
Check (0N)	–	0.37c	–	–	0.120b	0.43d	–	–	0.214c
Urea	–	0.99a	0.62a	0.85a	0.264a	0.83b	0.40b	0.50b	0.314b
Urea	PCU	1.17a	0.80a	1.12a	0.322a	1.17a	0.74a	0.95a	0.451a
Urea	UNI	0.63b	0.26b	0.38a	0.172b	0.76bc	0.32bc	0.42bc	0.312b
Urea	NI	0.46bc	0.09b	0.10a	0.133b	0.53cd	0.10c	0.12c	0.248bc
Urea	DUI	1.01a	0.64a	0.88a	0.269a	0.76bc	0.33bc	0.42bc	0.293bc
		**Spring applied**							
		**2016–2017**				**2017–2018**			
Check (0N)	–	0.44 c	–	–	0.139c	0.62b	–	–	0.311bc
Urea	–	1.22a	0.78a	1.05a	0.319a	1.20a	0.58a	0.75a	0.492ab
Urea	PCU	0.86b	0.43b	0.60ab	0.229b	1.40a	0.78a	0.98a	0.564a
Urea	UNI	0.56c	0.12bc	0.28bc	0.150c	0.79b	0.16b	0.22b	0.270bc
Urea	NI	0.44c	0.00c	0.03c	0.116c	0.66b	0.04b	0.05b	0.242c
Urea	DUI	0.62bc	0.18bc	0.15bc	0.158bc	1.25a	0.62a	0.80a	0.467abc

*Note*: For each application timing (i.e., fall and spring) and within columns, means followed by the same letter are not significantly different. Means separation was performed using the LSD test at a significance level of 0.05.

Abbreviations: EENF, enhanced efficiency nitrogen fertilizer; DUI, dual urease inhibitor; NI, nitrification inhibitor; PCU, polymer‐coated urea; UNI, urease and nitrification inhibitor.

^a^ Annual cumulative N_2_O emissions (kg N_2_O‐N ha^−1^).

^b^ Fertilizer‐induced emissions (FIEs; kg N_2_O‐N ha^−1^) = t‐N_2_O‐treatment − t‐N_2_O‐check; nitrous oxide emission factor (EF; %) = 100*(FIE/Nr), where Nr is the N application rate (kg N ha^−1^) and YSE (kg N_2_O‐N Mg^−1^ grain).

Fertilizer‐induced N_2_O emissions generally followed the trend: PCU = Urea > DUI > UNI > NI, with significant and consistent reductions in emissions observed when using the NI and DUI. On average, the use of the NI and UNI reduced FIEs by 63% and 91%, respectively. In comparison, stabilized urea containing only a urease inhibitor (i.e., the DUI) reduced cumulative FIEs by an average of 25%, although this reduction was statistically significant (*p* < 0.005) in only one of four N‐years: specifically, following the Spring 2016 application. In contrast, the PCU produced FIEs that were approximately 15% higher than those from conventional urea, although significant reductions relative to urea were observed following the Spring 2016 N application (Table 2).

In 2016, YSEs from the unfertilized check plots averaged 0.12 and 0.14 kg N_2_O‐N Mg^−^^1^ grain for the fall and spring N applications, respectively (Table 2). In contrast, YSEs from plots treated with urea averaged 0.26 and 0.32 kg N_2_O‐N Mg^−^^1^ grain for the fall and spring applications, respectively. Following the fall application, only urea‐based products with an NI resulted in YSEs that were significantly lower than those from the urea‐treated plots and comparable to the check plots. Following spring application, however, all EENFs produced lower YSEs than urea.

In general, FIEs and YSEs were highest when N was applied as urea or PCU. The stabilized urea product containing only a urease inhibitor showed inconsistent performance, with significant reductions in FIEs and YSEs observed in just one of four N‐years (Table 2). In contrast, urea products combined with an NI consistently reduced emissions, with significant decreases in both FIEs and YSEs across all four N‐years. Notably, the NI achieved the greatest reductions, lowering FIEs by 75%–99% and YSEs by 20%–50% compared to urea.

Aside from the fall application in Year 1F, NIs consistently reduced EFs relative to urea (*p* < 0.05). In particular, the NI had an EF 97% lower than conventional urea when spring‐applied in Year 1S. In contrast, the PCU generally had a comparable or an increased EF relative to urea (Table 2).

### 3.5 Crop yields

Grain yields in 2016 ranged from 3.48 to 3.93 Mg ha^−1^ for the plots receiving fertilizer N and 3.12 Mg ha^−1^ for the check plots (Table 2). Yield differences among the plots receiving N fertilizer were not significant (*p* > 0.05). Substantial hail damage to the crop in the late summer of 2017 prevented collection of grain yields; however, biomass yields were used to calculate estimated grain yields. Estimated grain yields for the fertilized plots averaged 2.62 ± 0.2 Mg ha^−1^ for the fall applications and 2.82 ± 0.2 Mg ha^−1^ for the spring applications.

## 4 DISCUSSION

### 4.1 Influence of fall‐applied fertilizer on N_2_O emissions in the Canadian Prairies

Emissions of N_2_O from soil are highly variable in time and space, occurring in “hot spots” and “hot moments” wherein the most intense emissions are associated with some sort of triggering event (Wagner‐Riddle et al., 2020; Yates et al., 2006). Primary triggering events include snow melt accompanying an increase in temperature during the spring thaw (Flesch et al., 2018; Wagner‐Riddle et al., 2017), precipitation events occurring shortly after the application of N fertilizer (Miller et al., 2022), and large, early‐ to mid‐season rainfall events that can trigger denitrification (Daly et al., 2022; Thilakarathna et al., 2020). In contrast, late‐season rainfall events generally result in minimal N_2_O emissions, as the soil N pool has typically been depleted by the crop (Chai et al., 2020; Thilakarathna et al., 2020). Critically, triggering events interact with soil conditions and N fertilizer timing, exerting a strong influence on the magnitude of N_2_O emissions and complicating the ability to draw conclusions regarding the impact of application timing alone (Burton et al., 2008; Thilakarathna et al., 2020). However, a trend of reduced N_2_O emissions from spring fertilizer application relative to fall is evident in research on cereal and canola crops in the Canadian Prairies (Burton et al., 2008; Hao et al., 2001; Lemke et al., 2006; Lin et al., 2017; Soon et al., 2011). Our findings suggest that rather than uniformly increasing or decreasing emissions, altering N fertilizer timing shifts the period of peak N_2_O loss from predominantly during spring thaw following fall application, to the early growing season following spring application (Figures 3 and 4). This temporal shift raises important questions about how best to target and implement mitigation strategies.

In agreement with Tenuta et al. (2016), N fertilizer application in the fall did not result in significant N_2_O emissions immediately post‐application during the fall, despite the increased precipitation during the fall over the study period. However, emissions observed during the spring thaw following fall‐applied N in Year 1F (2016) accounted for most annual emissions (Figure 3). Further, while spring thaw emissions in Year 2 were comparatively lower even though fall precipitation, and probably antecedent soil moisture, was greater than the long‐term average, it is probable that spring thaw emissions in Year 2F (2017) were underestimated. This underestimation is due to several short freeze‐thaw events (lasting four to six days) that occurred three times prior to the final thaw in mid‐March, which were not captured.

A broad body of literature supports our findings that N_2_O emissions are minimal during the late fall and winter after N application in regions where air and surface soil temperatures remain below freezing for months at a time but increase with the onset of spring thaw (Asgedom et al., 2014; Chantigny et al., 2019; Tenuta et al., 2016; Wood et al., 2024). However, while soil N transformations leading to N_2_O production and release may be slowed during winter months, microbial survival and activity are preserved within liquid water films in soil even under freezing conditions (Clark et al., 2009; Congreves et al., 2018). This is particularly evident in soils with high clay content, such as those at our study site, and in regions where microbial communities are well‐adapted to prolonged winter conditions (Chantigny et al., 2019; Congreves et al., 2018).

Evidence of sustained microbial activity during freezing temperatures is supported by data from the PRS probes, which recorded continued release of NH_4_^+^ from fall‐fertilized treatments throughout the winter burial period in Year 2F despite frozen soil conditions and then a substantial increase in NO_3_^−^ supply rates across all plots during the spring thaw (Figure 2D). To our knowledge, this study represents the first application of in situ ion exchange resins over winter in Western Canada to measure soil N fluxes over time, rather than relying on static measurements of soil N. PRS probe measurements are influenced by soil temperature and soil moisture, as ion diffusion to the resin surface becomes increasingly restricted under dry conditions, potentially confounding interpretation (Qian & Schoenau, 2002). Nevertheless, the observed patterns were consistent with the expected modes of action of the EENFs evaluated. Although the PRS probe findings did not directly correspond with soil mineral N measurements (Figures S3 and S4), this was expected because the methods quantify different aspects of soil N dynamics. PRS probes integrate cumulative inorganic N supply over the deployment period, whereas conventional soil extractions provide a single‐time‐point measurement of soil mineral N concentration (Harrison & Maynard, 2014). Thus, the two methods are related, but not directly comparable.

Wagner‐Riddle et al. (2024) concluded that elevated fall NO_3_^−^ concentrations are strong positive predictors of nongrowing season N_2_O emissions and reducing fall soil NO_3_^−^ levels is an effective mitigation strategy. However, our findings suggest this relationship, and thus mitigation strategies, may be more nuanced in fall‐fertilized agroecosystems where soil microbial communities are adapted to persist under extreme winter conditions (Chantigny et al., 2019; Clark et al., 2009). Ideally, consideration should be paid to multiple soil N pools in the fall season to implement strategies to reduce nongrowing season N losses. This includes soil NH_4_^+^ concentrations, which can undergo nitrification over the winter months, leading to the accumulation of NO_3_^−^ in the soil and creating a pool of available N for rapid denitrification during spring thaw (Chantigny et al., 2019; Tenuta et al., 2016). This dynamic is of particular importance in annual cropping systems dominated by spring‐seeded crops, which are prevalent across Western Canada (Agriculture and Agri‐Food Canada, 2024; Lassoued et al., 2025), as there is minimal N uptake by plants during the nongrowing season to mitigate these losses.

### 4.2 EENFs are effective at reducing N_2_O emissions from N fertilizer applications in the Canadian Prairies

The use of EENFs substantially reduced spring thaw‐induced and cumulative annual N_2_O emissions from fall‐applied fertilizers (Figure 3; Table S3). In particular, reductions were observed with stabilized N products containing NIs, which suppress the activity of nitrifying bacteria, even under freezing soil conditions, by delaying the oxidation of NH_4_^+^ to NO_3_^−^ (Chantigny et al., 2019; Ruser & Schulz, 2015). Notably, following fall urea application in Year 1F, spring thaw emissions represented 60%–80% of total annual N_2_O losses; however, applying an NI either alone or in combination with a UNI reduced spring thaw emissions by up to 50%.

Importantly, the efficacy of EENFs at reducing N_2_O emissions was enhanced when fertilizers were applied in the spring. Across treatments, the greatest emission reductions were consistently observed with spring applications, irrespective of the magnitude of emissions. This enhanced efficacy may be due to moderate increases in soil temperature during spring, which have been shown to improve the performance of NIs in some instances (Menendez et al., 2012). Additionally, it may reflect better alignment between fertilizer application and crop N uptake, thereby limiting the pool of N substrates available for nitrification and denitrification (Flesch et al., 2018; Wagner‐Riddle et al., 2017). In contrast, fall fertilizer applications for spring‐seeded crops result in a period between N availability and crop uptake, increasing the risk of N accumulation and subsequent losses, particularly during spring thaw events (Daly et al., 2022).

The DUI yielded significant emissions reductions only when applied in the spring and only during one growing season. The lack of consistent reduction is in line with previous research (Akiyama et al., 2010; Wood et al., 2024) and likely due to the fact that urease inhibitors do not have a direct effect on the N_2_O production processes of nitrification or denitrification. Instead, urease inhibitors slow the hydrolysis of urea into ammonia (NH_3_), thereby reducing NH_3_ volatilization and the potential for associated N losses (Cantarella et al., 2018; Hu et al., 2020; Rawluk et al., 2001). Critically, reductions in NH_3_ losses may mitigate indirect N_2_O emissions (Wang et al., 2020; Wu et al., 2021; Wulf et al., 2002). Although NH_3_ emissions were not measured in this study, they represent an opportunity for future research, as few studies in Western Canada have investigated the wider impact of EENFs on indirect N_2_O emissions.

The PCU was the least effective at reducing N_2_O emissions relative to urea, with greater cumulative emissions, FIE, and a higher EF in three of four N‐years (Table 2). Previous research has shown that PCU products have increased soil N availability later in the growing season when crop N demand has slowed, thereby delaying fertilizer‐induced N_2_O fluxes. This delayed release can offset the N_2_O reductions achieved earlier in the growing season and can even result in higher cumulative N_2_O emissions compared to urea (An et al., 2020; Li et al., 2012). This pattern is consistent with our findings; following spring applications in particular, the N_2_O emission pulse associated with the PCU during the growing season was prolonged and occurred later compared to the other EENFs (Figure 1B).

### 4.3 Future research to support EENF adoption on the Canadian Prairies

Consistent with Wood et al. (2024) and Thilakarathna et al. (2020), EENFs did not significantly affect grain yield compared to urea in either growing season, despite differences in cumulative N_2_O emission. In theory, EENFs should require lower N application rates than conventional urea due to reduced N losses and improved N use efficiency (NUE) (Fan et al., 2022; Ferland et al., 2024; Rose et al., 2018). In our experiment, we applied EENFs at the same N rate as conventional fertilizers to avoid the confounding impact of lower N fertilizer rates on N_2_O emissions. However, future studies should test whether lower EENF rates can sustain equivalent yields while mitigating N_2_O emissions (Fan et al., 2022; Ferland et al., 2024; Rose et al., 2018). Given their higher cost, applying EENFs at up to 30% lower rates may be more economical, bridging the price gap between EENFs and conventional fertilizer products and supporting broader adoption (Ferland et al., 2024; Tenuta et al., 2023).

Additional complexity arises when considering the effects of climate change on temperature and precipitation patterns in the Canadian Prairies, the latter of which are historically highly variable and subject to considerable uncertainty in future climate projections (Bonsal et al., 2013; Mapfumo et al., 2023). Our study period was characterized by above‐average precipitation and deviations in precipitation patterns relative to the 30‐year climate normals. As climate change is expected to alter the frequency and intensity of temperature and precipitation extremes in the Canadian Prairies, observations collected during outlier years that elucidate how EENF performance responds to environmental extremes are becoming increasingly important. Such datasets are critical for the validation and calibration of process‐based models, including the Canadian version of the DeNitrification–DeComposition Model (DNDC v.CAN), which relies on robust empirical observations to accurately represent soil–plant–atmosphere interactions (Dutta et al., 2017).

## 5 CONCLUSIONS

This study demonstrates that EENFs can significantly reduce N_2_O emissions from rainfed cropping systems in Saskatchewan, particularly stabilized N products containing an NI. Products with an NI alone = or combined with a UNI consistently produced the greatest emissions reductions, regardless of application timing. In contrast, the DUI showed inconsistent results, with significant reductions observed only in 1 year and only with spring application. The PCU was the least effective overall. Across all treatments, spring applications led to the greatest N_2_O emissions reductions. Future research should investigate the effects of EENFs on indirect N_2_O emissions and determine whether reduced EENF application rates can maintain crop yields comparable to those achieved with conventional fertilizer rates under Canadian Prairie conditions.

## AUTHOR CONTRIBUTIONS

**Erin Daly**: Formal analysis; visualization; writing—original draft; writing—review and editing. **Cheyne Ogilvie**: Conceptualization; data curation; formal analysis; investigation; project administration; writing—original draft. **Reynald L. Lemke**: Conceptualization; funding acquisition; investigation; methodology; project administration; resources; supervision; writing—review and editing. **Richard E. Farrell**: Conceptualization; data curation; formal analysis; funding acquisition; investigation; methodology; project administration; resources; software; supervision; validation; visualization; writing—original draft; writing—review and editing.

## CONFLICT OF INTEREST STATEMENT

The authors declare no conflicts of interest.

## Supporting information



The supplemental materials include schematics of the experimental setup, a summary of field management activities, ANOVA results from the three‐way model of cumulative N_2_O emissions, a seasonal breakdown of emissions with corresponding statistical analyses, and soil NH_4_^+^ and NO_3_^−^ concentrations of surface soil samples.

## References

[jeq270251-bib-0001] Abalos, D. , Smith, W. N. , Grant, B. B. , Drury, C. F. , MacKell, S. , & Wagner‐Riddle, C. (2016). Scenario analysis of fertilizer management practices for N_2_O mitigation from corn systems in Canada. Science of the Total Environment, 573, 356–365. 27572528 10.1016/j.scitotenv.2016.08.153

[jeq270251-bib-0003] Agriculture and Agri‐Food Canada . (2024). Annual crop inventory . Government of Canada. https://open.canada.ca/data/en/dataset/ba2645d5‐4458‐414d‐b196‐6303ac06c1c9

[jeq270251-bib-0081] Akiyama, H. , Yan, X. , & Yagi, K. (2010). Evaluation of effectiveness of enhanced‐efficiency fertilizers as mitigation options for N2O and NO emissions from agricultural soils: meta‐analysis. Global Change Biology, 16(6), 1837–1846.

[jeq270251-bib-0004] Amadi, C. C. , Van Rees, K. C. , & Farrell, R. E. (2016). Soil–atmosphere exchange of carbon dioxide, methane and nitrous oxide in shelterbelts compared with adjacent cropped fields. Agriculture, Ecosystems & Environment, 223, 123–134.

[jeq270251-bib-0005] An, H. , Owens, J. , Stoeckli, J. , Hao, X. , Beres, B. , & Li, Y. (2020). Nitrous oxide emissions following split fertilizer application on winter wheat grown on Mollisols of Southern Alberta, Canada. Geoderma Regional, 21, e00272.

[jeq270251-bib-0078] Asgedom, H. , Tenuta, M. , Flaten, D. N. , Gao, X. , & Kebreab, E. (2014). Nitrous oxide emissions from a clay soil receiving granular urea formulations and dairy manure. Agronomy Journal, 106(2), 732–744. 10.2134/agronj2013.0096

[jeq270251-bib-0006] Awada, L. , Nagy, C. , & Phillips, P. W. (2021). Contribution of land use practices to GHGs in the Canadian Prairies crop sector. PLoS ONE, 16(12), e0260946. 34919544 10.1371/journal.pone.0260946 PMC8682883

[jeq270251-bib-0007] Bonsal, B. R. , Aider, R. , Gachon, P. , & Lapp, S. (2013). An assessment of Canadian Prairie drought: Past, present, and future. Climate Dynamics, 41(2), 501–516.

[jeq270251-bib-0008] Burton, D. L. , Li, X. , & Grant, C. A. (2008). Influence of fertilizer nitrogen source and management practice on N_2_O emissions from two Black Chernozemic soils. Canadian Journal of Soil Science, 88(2), 219–227.

[jeq270251-bib-0010] Cantarella, H. , Otto, R. , Soares, J. R. , & Silva, A. G. D. B. (2018). Agronomic efficiency of NBPT as a urease inhibitor: A review. Journal of advanced research, 13, 19–27. 30094079 10.1016/j.jare.2018.05.008 PMC6077139

[jeq270251-bib-0011] Chai, L. L. , Hernandez‐Ramirez, G. , Dyck, M. , Pauly, D. , Kryzanowski, L. , Middleton, A. , Powers, L.‐A. , Lohstraeter, G. , & Werk, D. (2020). Can fertigation reduce nitrous oxide emissions from wheat and canola fields? Science of the Total Environment, 745, 141014. 32758754 10.1016/j.scitotenv.2020.141014

[jeq270251-bib-0079] Chantigny, M. H. , Bittman, S. , Larney, F. J. , Lapen, D. , Hunt, D. E. , Goyer, C. , & Angers, D. A. (2019). A multi‐region study reveals high overwinter loss of fall‐applied reactive nitrogen in cold and frozen soils. Canadian Journal of Soil Science, 99(2), 126–135.

[jeq270251-bib-0012] Clark, K. , Chantigny, M. H. , Angers, D. A. , Rochette, P. , & Parent, L. É. (2009). Nitrogen transformations in cold and frozen agricultural soils following organic amendments. Soil Biology and Biochemistry, 41(2), 348–356.

[jeq270251-bib-0080] Congreves, K. A. , Wagner‐Riddle, C. , Si, B. C. , & Clough, T. J. (2018). Nitrous oxide emissions and biogeochemical responses to soil freezing‐thawing and drying‐wetting. Soil Biology and Biochemistry, 117, 5–15.

[jeq270251-bib-0013] Daly, E. , Kim, K. , Hernandez‐Ramirez, G. , & Flesch, T. (2022). Perennial grain crops reduce N_2_O emissions under specific site conditions. Agriculture, Ecosystems & Environment, 326, 107802.

[jeq270251-bib-0014] David, C. , Lemke, R. , Helgason, W. , & Farrell, R. E. (2018). Current inventory approach overestimates the effect of irrigated crop management on soil‐derived greenhouse gas emissions in the semi‐arid Canadian Prairies. Agricultural Water Management, 208, 19–32.

[jeq270251-bib-0015] DeBruin, J. L. , Gorowara, R. L. , Schussler, J. , Pape, R. , Grafton, M. , Liu, L. U. , Macchia, J. , Kendra, S. , Zhang, J. , & Burch, R. (2021). Evaluating polymer‐coated fertilizer prototypes designed for planting along with maize seed. Agronomy Journal, 113(2), 1619–1639.

[jeq270251-bib-0017] Dimkpa, C. O. , Fugice, J. , Singh, U. , & Lewis, T. D. (2020). Development of fertilizers for enhanced nitrogen use efficiency—Trends and perspectives. Science of the Total Environment, 731, 139113. 32438083 10.1016/j.scitotenv.2020.139113

[jeq270251-bib-0018] Drury, C. F. , Yang, X. , Reynolds, W. D. , Calder, W. , Oloya, T. O. , & Woodley, A. L. (2017). Combining urease and nitrification inhibitors with incorporation reduces ammonia and nitrous oxide emissions and increases corn yields. Journal of Environmental Quality, 46(5), 939–949. 28991976 10.2134/jeq2017.03.0106

[jeq270251-bib-0019] Dutta, B. , Grant, B. B. , Campbell, C. A. , Lemke, R. L. , Desjardins, R. L. , & Smith, W. N. (2017). A multi model evaluation of long‐term effects of crop management and cropping systems on nitrogen dynamics in the Canadian semi‐arid prairie. Agricultural Systems, 151, 136–147.

[jeq270251-bib-0020] ECCC . (2024). National inventory report 1990–2022: Greenhouse gas sources and sinks in Canada . Government of Canada. https://publications.gc.ca/collections/collection_2024/eccc/En81‐4‐2022‐1‐eng.pdf

[jeq270251-bib-0021] Engel, R. , Liang, D. L. , Wallander, R. , & Bembenek, A. (2010). Influence of urea fertilizer placement on nitrous oxide production from a silt loam soil. Journal of Environmental Quality, 39(1), 115–125. 20048299 10.2134/jeq2009.0130

[jeq270251-bib-0076] Environment Canada . (2022). Historical data. Environment Canada. https://climate.weather.gc.ca/historical_data/search_historic_data_e.html

[jeq270251-bib-0073] Fan, D. , He, W. , Smith, W. N. , Drury, C. F. , Jiang, R. , Grant, B. B. , Shi, Y. , Song, D. , Chen, Y. , Wang, X. , He, P. , & Zou, G. (2022). Global evaluation of inhibitor impacts on ammonia and nitrous oxide emissions from agricultural soils: A meta‐analysis. Global Change Biology, 28(17), 5121–5141. 10.1111/gcb.16294 35678108

[jeq270251-bib-0023] Fast, A. , Strydhorst, S. , Wang, Z. , Hernandez‐Ramirez, G. , Hao, X. , Semach, G. , Thompson, L. , Holzapfel, C. , Enns, J. , Spaner, D. , & Beres, B. L. (2023). Integrating enhanced efficiency fertilizers and nitrogen rates to improve Canada Western Red Spring wheat. Canadian Journal of Plant Science, 104(2), 144–160.

[jeq270251-bib-0024] Ferland, D. , Wagner‐Riddle, C. , Brown, S. E. , Bourgault, M. , Helgason, W. , Farrell, R. E. , & Congreves, K. A. (2024). Improved nitrogen fertilizer management reduces nitrous oxide emissions in a northern Prairie cropland. Science of the Total Environment, 956, 177211. 39481573 10.1016/j.scitotenv.2024.177211

[jeq270251-bib-0025] Fertilizer Canada . (2022). 4R nutrient stewardship grower adoption across Canada . https://fertilizercanada.ca/wp‐content/uploads/2022/08/SPARK‐FERTILIZER‐USE‐IN‐CANADA‐REPORT‐2022‐VF_08_04_2022.pdf

[jeq270251-bib-0026] Flesch, T. K. , Baron, V. S. , Wilson, J. D. , Basarab, J. A. , Desjardins, R. L. , Worth, D. , & Lemke, R. L. (2018). Micrometeorological measurements reveal large nitrous oxide losses during spring thaw in Alberta. Atmosphere, 9(4), 128.

[jeq270251-bib-0027] Forster, P. , Storelvmo, T. , Armour, K. , Collins, W. , Dufresne, J. L. , Frame, D. , Lunt, D. , Mauritsen, T. , Palmer, M. , Watanabe, M. , & Wild, M. (2021). The Earth's energy budget, climate feedbacks, and climate sensitivity. In V. Masson‐Delmotte , P. Zhai , A. Pirani , S. L. Connors , C. Péan , S. Berger , N. Caud , Y. Chen , L. Goldfarb , M. I. Gomis , M. Huang , K. Leitzell , E. Lonnoy , J. B. R. Matthews , T. K. Maycock , T. Waterfield , O. Yelekçi , R. Yu , & B. Zhou (Eds.), Climate change 2021: The physical science basis. Contribution of working group I to the sixth assessment report of the intergovernmental panel on climate change (pp. 923–1054). Cambridge University Press,.

[jeq270251-bib-0028] Gao, X. , Asgedom, H. , Tenuta, M. , & Flaten, D. N. (2015). Enhanced efficiency urea sources and placement effects on nitrous oxide emissions. Agronomy Journal, 107, 265–277. 10.2134/agronj14.0213

[jeq270251-bib-0029] Halvorson, A. D. , Snyder, C. S. , Blaylock, A. D. , & Del Grosso, S. J. (2014). Enhanced‐efficiency nitrogen fertilizers: Potential role in nitrous oxide emission mitigation. Agronomy Journal, 106(2), 715–722.

[jeq270251-bib-0030] Hao, X. , Chang, C. , Carefoot, J. M. , Janzen, H. H. , & Ellert, B. H. (2001). Nitrous oxide emissions from an irrigated soil as affected by fertilizer and straw management. Nutrient Cycling in Agroecosystems, 60, 1–8.

[jeq270251-bib-0031] Harrison, D. J. , & Maynard, D. G. (2014). Nitrogen mineralization assessment using PRS™ probes (ion‐exchange membranes) and soil extractions in fertilized and unfertilized pine and spruce soils. Canadian Journal of Soil Science, 94(1), 21–34.

[jeq270251-bib-0032] Hu, Y. , Gaßner, M. P. , Weber, A. , Schraml, M. , & Schmidhalter, U. (2020). Direct and indirect effects of urease and nitrification inhibitors on N_2_O‐N losses from urea fertilization to winter wheat in Southern Germany. Atmosphere, 11(8), 782.

[jeq270251-bib-0075] Hutchinson, G. L. , & Mosier, A. R. (1981). Improved soil cover method for field measurement of nitrous oxide fluxes. Soil Science Society of America Journal, 45(2), 311–316. 10.2136/sssaj1981.03615995004500020017x

[jeq270251-bib-0033] Kalra, Y. , Crumbaugh, J. , & Maynard, D. (2007). Nitrate and exchangeable ammonium nitrogen. In M. R. Carter & E. G. Gregorich (Eds.), Soil sampling and methods of analysis (2nd ed., pp. 71–88). CRC Press.

[jeq270251-bib-0034] Kanter, D. R. , Zhang, X. , & Mauzerall, D. L. (2015). Reducing nitrogen pollution while decreasing farmers’ costs and increasing fertilizer industry profits. Journal of Environmental Quality, 44(2), 325–335. 26023952 10.2134/jeq2014.04.0173

[jeq270251-bib-0035] Lassoued, F. , Slade, P. , & Dyck, A. (2025). Crop rotations and canola yields: Evidence from field‐level data in Western Canada. Agronomy Journal, 117(1), e21739.

[jeq270251-bib-0036] Lemke, R. , Malhi, S. S. , Lafond, G. , Brandt, S. A. , & Farrell, R. E. (2006). Fertilizer‐N management and nitrous oxide emissions from four sites in Saskatchewan. University of Saskatchewan. https://hdl.handle.net/10388/9459

[jeq270251-bib-0037] Li, C. , Hao, X. , Blackshaw, R. E. , O'Donovan, J. T. , Harker, K. N. , & Clayton, G. W. (2012). Nitrous oxide emissions in response to ESN and urea, herbicide management and canola cultivar in a no‐till cropping system. Soil and Tillage Research, 118, 97–106.

[jeq270251-bib-0038] Lin, S. , Hernandez‐Ramirez, G. , Kryzanowski, L. , Wallace, T. , Grant, R. , Degenhardt, R. , Berger, N. , Lohstraeter, G. , & Powers, L.‐A. (2017). Timing of manure injection and nitrification inhibitors impacts on nitrous oxide emissions and nitrogen transformations in a barley crop. Soil Science Society of America Journal, 81(6), 1595–1605.

[jeq270251-bib-0039] Mapfumo, E. , Chanasyk, D. S. , Puurveen, D. , Elton, S. , & Acharya, S. (2023). Historic climate change trends and impacts on crop yields in key agricultural areas of the prairie provinces in Canada: A literature review. Canadian Journal of Plant Science, 103(3), 243–258.

[jeq270251-bib-0040] Menéndez, S. , Barrena, I. , Setien, I. , González‐Murua, C. , & Estavillo, J. M. (2012). Efficiency of nitrification inhibitor DMPP to reduce nitrous oxide emissions under different temperature and moisture conditions. Soil Biology and Biochemistry, 53, 82–89.

[jeq270251-bib-0041] Miller, L. T. , Griffis, T. J. , Erickson, M. D. , Turner, P. A. , Deventer, M. J. , Chen, Z. , Yu, Z. , Venterea, R. T. , Baker, J. M. , & Frie, A. L. (2022). Response of nitrous oxide emissions to individual rain events and future changes in precipitation. Journal of Environmental Quality, 51(3), 312–324. 35357715 10.1002/jeq2.20348

[jeq270251-bib-0045] Owens, J. , An, H. , Beres, B. , Mohr, R. , & Hao, X. (2019). The effects of split application of enhanced efficiency fertilizers on non‐winter nitrous oxide emissions from winter wheat. Canadian Journal of Soil Science, 100(1), 26–43.

[jeq270251-bib-0046] Pedersen, A. R. , Petersen, S. O. , & Schelde, K. (2010). A comprehensive approach to soil‐atmosphere trace‐gas flux estimation with static chambers. European Journal of Soil Science, 61(6), 888–902.

[jeq270251-bib-0047] Phan, T. (2022). Can enhanced efficiency N fertilizers mitigate N_2_O emissions without compromising crop yield? [Master's thesis, University of Saskatchewan].

[jeq270251-bib-0048] Qian, P. , & Schoenau, J. J. (2002). Practical applications of ion exchange resins in agricultural and environmental soil research. Canadian Journal of Soil Science, 82(1), 9–21.

[jeq270251-bib-0049] R Development Core Team . (2011). R: A language and environment for statistical computing . R Foundation for Statistical Computing. www.r‐project.org

[jeq270251-bib-0050] Ravishankara, A. R. , Daniel, J. S. , & Portmann, R. W. (2009). Nitrous oxide (N_2_O): The dominant ozone‐depleting substance emitted in the 21st century. Science, 326(5949), 123–125. 19713491 10.1126/science.1176985

[jeq270251-bib-0051] Rawluk, C. D. L. , Grant, C. A. , & Racz, G. J. (2001). Ammonia volatilization from soils fertilized with urea and varying rates of urease inhibitor NBPT. Canadian Journal of Soil Science, 81(2), 239–246.

[jeq270251-bib-0082] Rose, T. J. , Wood, R. H. , Rose, M. T. , & Van Zwieten, L. (2018). A re‐evaluation of the agronomic effectiveness of the nitrification inhibitors DCD and DMPP and the urease inhibitor NBPT. Agriculture, Ecosystems & Environment, 252, 69–73.

[jeq270251-bib-0054] Ruser, R. , & Schulz, R. (2015). The effect of nitrification inhibitors on the nitrous oxide (N2O) release from agricultural soils—A review. Journal of Plant Nutrition and Soil Science, 178(2), 171–188.

[jeq270251-bib-0055] Soon, Y. K. , Malhi, S. S. , Lemke, R. L. , Lupwayi, N. Z. , & Grant, C. A. (2011). Effect of polymer‐coated urea and tillage on the dynamics of available N and nitrous oxide emission from Gray Luvisols. Nutrient Cycling in Agroecosystems, 90, 267–279.

[jeq270251-bib-0056] Statistics Canada . (2025). Table: 32‐10‐0038‐01: Fertilizer shipments to Canadian agriculture and export markets, by product type and fertilizer year, cumulative data . https://www150.statcan.gc.ca/n1/en/catalogue/3210003801

[jeq270251-bib-0072] Tenuta, M. , Gao, X. , Tiessen, K. H. , Baron, K. , & Sparling, B. (2023). Placement and nitrogen source effects on N2O emissions for canola production in Manitoba. Agronomy Journal, 115(5), 2369–2383.

[jeq270251-bib-0077] Tenuta, M. , Gao, X. , Flaten, D. N. , & Amiro, B. D. (2016). Lowernitrous oxide emissions from anhydrous ammonia application prior to soil freezing in late fall than spring pre‐plant application. Journal of Environmental Quality, 45(4), 1133–1143. 10.2134/jeq2015.03.0159 27380060

[jeq270251-bib-0058] Thilakarathna, S. K. , Hernandez‐Ramirez, G. , Puurveen, D. , Kryzanowski, L. , Lohstraeter, G. , Powers, L.‐A. , Quan, N. , & Tenuta, M. (2020). Nitrous oxide emissions and nitrogen use efficiency in wheat: Nitrogen fertilization timing and formulation, soil nitrogen, and weather effects. Soil Science Society of America Journal, 84(6), 1910–1927.

[jeq270251-bib-0074] Verburg, K. , Thorburn, P. J. , Vilas, M. P. , Biggs, J. S. , Zhao, Z. , & Bonnett, G. D. (2022). Why are the benefits of enhanced‐efficiency fertilizers inconsistent in the field? Prerequisite conditions identified from simulation analyses: Why are the benefits of enhanced‐efficiency fertilizers inconsistent in the field? Prerequisite conditions identified from simulation analyses. Agronomy for Sustainable Development, 42(4), 73.

[jeq270251-bib-0060] Wagner‐Riddle, C. , Baggs, E. M. , Clough, T. J. , Fuchs, K. , & Petersen, S. O. (2020). Mitigation of nitrous oxide emissions in the context of nitrogen loss reduction from agroecosystems: Managing hot spots and hot moments. Current Opinion in Environmental Sustainability, 47, 46–53.

[jeq270251-bib-0061] Wagner‐Riddle, C. , Congreves, K. A. , Abalos, D. , Berg, A. A. , Brown, S. E. , Ambadan, J. T. , Gao, X. , & Tenuta, M. (2017). Globally important nitrous oxide emissions from croplands induced by freeze–thaw cycles. Nature Geoscience, 10(4), 279–283.

[jeq270251-bib-0062] Wagner‐Riddle, C. , Congreves, K. A. , Brown, S. E. , Helgason, W. D. , & Farrell, R. E. (2024). Overwinter and spring thaw nitrous oxide fluxes in a northern Prairie cropland are limited but a significant proportion of annual emissions. Global Biogeochemical Cycles, 38(4), e2023GB008051.

[jeq270251-bib-0063] Wagner‐Riddle, C. , & Thurtell, G. W. (1998). Nitrous oxide emissions from agricultural fields during winter and spring thaw as affected by management practices. Nutrient Cycling in Agroecosystems, 52(2), 151–163.

[jeq270251-bib-0064] Wang, H. , Köbke, S. , & Dittert, K. (2020). Use of urease and nitrification inhibitors to reduce gaseous nitrogen emissions from fertilizers containing ammonium nitrate and urea. Global Ecology and Conservation, 22, e00933.

[jeq270251-bib-0065] Wilton, B. , Kapitan, K. , Gal, A. , Deveau, J. , Stoddart, H. , Lika, E. , & Zafiriou, M. (2024). From education to action: A review of greenhouse gas tools in pursuit of net‐zero agriculture. The Canadian Agri‐Food Policy Institute (CAPI).

[jeq270251-bib-0066] Wood, M. D. , Gao, X. , Tiessen, K. H. , Tenuta, M. , & Flaten, D. N. (2024). Enhanced efficiency urea fertilizers and timing effects on N_2_O emissions from spring wheat production in Manitoba. Agronomy Journal, 116(1), 51–72.

[jeq270251-bib-0067] Woodley, A. L. , Drury, C. F. , Yang, X. Y. , Phillips, L. A. , Reynolds, D. W. , Calder, W. , & Oloya, T. O. (2020). Ammonia volatilization, nitrous oxide emissions, and corn yields as influenced by nitrogen placement and enhanced efficiency fertilizers. Soil Science Society of America Journal, 84(4), 1327–1341.

[jeq270251-bib-0068] Wu, D. , Zhang, Y. , Dong, G. , Du, Z. , Wu, W. , Chadwick, D. , & Bol, R. (2021). The importance of ammonia volatilization in estimating the efficacy of nitrification inhibitors to reduce N_2_O emissions: A global meta‐analysis. Environmental Pollution, 271, 116365. 33388681 10.1016/j.envpol.2020.116365

[jeq270251-bib-0069] Wulf, S. , Maeting, M. , & Clemens, J. (2002). Application technique and slurry co‐fermentation effects on ammonia, nitrous oxide, and methane emissions after spreading: II. Greenhouse gas emissions. Journal of Environmental Quality, 31(6), 1795–1801. 12469828 10.2134/jeq2002.1795

[jeq270251-bib-0070] Yates, T. T. , Si, B. C. , Farrell, R. E. , & Pennock, D. J. (2006). Probability distribution and spatial dependence of nitrous oxide emission: Temporal change in hummocky terrain. Soil Science Society of America Journal, 70(3), 753–762.

[jeq270251-bib-0071] Zhang, X. , Zou, T. , Lassaletta, L. , Mueller, N. D. , Tubiello, F. N. , Lisk, M. D. , Lu, C. , Conant, R. T. , Dorich, C. D. , Gerber, J. , Tian, H. , Bruulsema, T. , Maaz, T. M. , Nishina, K. , Bodirsky, B. L. , Popp, A. , Bouwman, L. , Beusen, A. , Chang, J. , … Davidson, E. A. (2021). Quantification of global and national nitrogen budgets for crop production. Nature Food, 2(7), 529–540. 37117677 10.1038/s43016-021-00318-5

