# Supplementary material for: An evaluation of the N2O emission reduction potential of enhanced efficiency nitrogen fertilizers and application timing in a semi‐arid cereal cropping system
Source: J Environ Qual. 2026 Sep 7;55(5):e70251. doi: 10.1002/jeq2.70251 (PMC13550706; doi:10.1002/jeq2.70251)
Supplement: Supplementary file 1 [file JEQ2-55-0-s001.docx]

**Supplemental Materials for “An evaluation of the N_2_O emission reduction potential of enhanced efficiency nitrogen fertilizers and application timing in a semi-arid cereal cropping system”**

Erin Daly^a*^, Cheyne Ogilvie^b, c^, Reynald L. Lemke^d^, and Richard E. Farrell^b^

^a^ University of Guelph, School of Environmental Sciences

^b^ University of Saskatchewan, Department of Soil Science

^c^ Present address: Richardson International

^d^ AAFC-Saskatoon Research and Development Centre

*^*^ Corresponding author: edaly02@uoguelph.ca*

Table S1. Site management summary for both experimental years.

| **Activity** | **Year 1 (2015-17)^a^** | | **Year 2 (2016-18)^a^** | |
| --- | --- | --- | --- | --- |
|  | **Management** | | | |
|  |  | *Date* |  | *Date* |
| **Seeding** |  |  |  |  |
| Crop | HRSW^b^ | 4-May-16 | HRSW | 12-May-17 |
| *Variety* | AC Carberry |  | AC Carberry |  |
| **Harvest** |  | 30-Aug-16 |  | 27-Jul-17 |
|  | **Crop Inputs** | | | |
| **Fertilizer Application** | *kg ha^-1^* | *Date* | *kg ha^-1^* | *Date* |
| N-Products |  |  |  |  |
| fall-applied | 73 (N) | 21-Oct-15 | 77 (N) | 26-Oct-16 |
| spring-applied | 73 (N) | 04-May-16 | 77 (N) | 11-May-17 |
| TSP^c^ | 17 (P) | 04-May-16 | 17 (P) | 11-May-17 |
| K_2_SO_4_^d^ | 15 (K); 6 (S) | 04-May-16 | 15 (K); 6 (S) | 11-May-17 |
| **Herbicide Application**^e^ |  |  |  |  |
| Badge® + Simplicity^TM^ |  | 04-May-16 |  | 11-May-17 |
| **Fungicide Application**^e^ |  |  |  |  |
| Prosaro® |  | 01-Jul-16 |  | 05-Jul-17 |

^a^	The N_2_O measurement year was defined as the time from N fertilizer application in Year 1 to just prior to N fertilizer placement in Year 2. Thus, for fall applied N-year 1 was from October 2015 to October 2016, while for Year 2 it was from October 2016 to October 2017; for the spring applied N-year 1 was from May 2016 to May 2017, while for Year 2 it was from May 2017 to May 2018.

^b^	Hard red spring wheat.

^c^	Triple Superphosphate [Ca(H_2_PO_4_)_2_] applied at 88 kg ha^-1^, equivalent to 17.6 kg P ha^-1^.

^d^	Potassium sulfate (K_2_SO_4_) applied at 34 kg ha^-1^_,_ equivalent to 15 kg K ha^-1^ and 6 kg S ha^-1^.

^e^	Badge® (bromoxynil & MCPA) and Simplicity™ (pyroxsulam, cloquintocet-methyl) were applied to combat Group 1 resistant wild oats. Prosaro® 250 EC (prothioconazole & tebuconazole) was applied to combat leaf disease and fusarium head blight.

Table S2. Analysis of variance output table for cumulative N_2_O emissions.

|  | **DF** | **Sum Sq** | **Mean Sq** | **F-Value** | **Pr(>F)** |
| --- | --- | --- | --- | --- | --- |
| Block | 3 | 713954 | 237985 | 6.0712 | 0.0009947 |
| Nyear | 1 | 441595 | 441595 | 11.2654 | 0.0012873 |
| Timing | 1 | 145471 | 145471 | 3.7111 | 0.058173 |
| Product | 5 | 6507421 | 1301484 | 33.2018 | < 2.2e-16 |
| Nyear:Timing | 1 | 614560 | 614560 | 15.6779 | 0.0001802 |
| Nyear:Product | 5 | 288378 | 57676 | 1.4713 | 0.2104487 |
| Timing:Product | 5 | 303079 | 60616 | 1.5464 | 0.1869935 |
| Nyear:Timing:Product | 5 | 496506 | 99301 | 2.5333 | 0.0365265 |
| Residuals | 69 | 2704741 | 39199 |  |  |

Table S3. Seasonal cumulative N_2_O emissions for all application timings and experimental years.

| **N Source** | **EENF Product** | **Seasonal Cumulative N_2_O emissions**  **(kg N_2_O-N ha^-1^)^a^** | | | **Seasonal Cumulative N_2_O emissions**  **(kg N_2_O-N ha^-1^)** | | |
| --- | --- | --- | --- | --- | --- | --- | --- |
|  |  | **Fall** | **Spring** | **Growing Season** | **Fall** | **Spring** | **Growing Season** |
|  |  | *Year 1F (Oct. 2016 – Oct. 2017)* | | | *Year 2F (Oct. 2015 – Oct. 2016)* | | |
| Check (0N) | – – – | 0.007 ± 0.003 | 0.127 ± 0.026 c | 0.213 ± 0.038 | 0.037 ± 0.004 | 0.105 ± 0.016 c | 0.29 ± 0.015 b |
| Urea | – – – | 0.028 ± 0.007 | 0.671 ± 0.081 a | 0.255 ± 0.019 | 0.064 ± 0.008 | 0.341 ± 0.051 a | 0.427 ± 0.07 ab |
| Urea | PCU | 0.016 ± 0.004 | 0.811 ± 0.159 a | 0.283 ± 0.014 | 0.063 ± 0.021 | 0.315 ± 0.024 a | 0.79 ± 0.189 a |
| Urea | UNI | 0.016 ± 0.005 | 0.361 ± 0.032 b | 0.235 ± 0.03 | 0.056 ± 0.007 | 0.251 ± 0.036 ab | 0.451 ± 0.074 ab |
| Urea | NI | 0.013 ± 0.002 | 0.205 ± 0.015 bc | 0.219 ± 0.007 | 0.037 ± 0.011 | 0.165 ± 0.024 bc | 0.325 ± 0.043 b |
| Urea | DUI | 0.022 ± 0.008 | 0.725 ± 0.101 a | 0.237 ± 0.017 | 0.06 ± 0.01 | 0.236 ± 0.032 abc | 0.47 ± 0.087 ab |
| ANOVA *p*-value |  |  |  |  |  |  |  |
| Fertilizer Source |  | *0.1458* | *<0.001* | *0.3978* | *0.351* | *<0.001* | *<0.05* |
|  |  | *Year 1S (May 2016 – May 2017)* | | | *Year 2S (May 2017 – May 2018)* | | |
| Check (0N) | – – – | 0.002 ± 0.000 | 0.205 ± 0.041 | 0.231 ± 0.037 c | 0.057 ± 0.008 | 0.332 ± 0.062 | 0.232 ± 0.016 b |
| Urea | – – – | 0.003 ± 0.001 | 0.257 ± 0.067 | 0.959 ± 0.231 a | 0.059 ± 0.007 | 0.47 ± 0.085 | 0.673 ± 0.112 a |
| Urea | PCU | 0.003 ± 0.001 | 0.213 ± 0.025 | 0.648 ± 0.117 ab | 0.072 ± 0.015 | 0.371 ± 0.04 | 0.955 ± 0.161 a |
| Urea | UNI | 0.003 ± 0.000 | 0.235 ± 0.051 | 0.325 ± 0.049 bc | 0.072 ± 0.012 | 0.433 ± 0.055 | 0.282 ± 0.024 b |
| Urea | NI | 0.003 ± 0.001 | 0.175 ± 0.049 | 0.258 ± 0.028 c | 0.061 ± 0.01 | 0.366 ± 0.057 | 0.23 ± 0.022 b |
| Urea | DUI | 0.003 ± 0.000 | 0.260 ± 0.041 | 0.356 ± 0.058 bc | 0.074 ± 0.017 | 0.469 ± 0.102 | 0.703 ± 0.187 a |
| ANOVA p value |  |  |  |  |  |  |  |
| Fertilizer Product |  | *0.632* | *0.7881* | *<0.01* | *0.8586* | *0.6317* | *<0.001* |

^a^ For each application timing (i.e., fall or spring) and year, within column means followed by the same letter are not significantly different. Means separation was performed using the LSD test at a significance level of 0.05.

Figure S1. Schematic showing the location of the experimental plots. In year 1 (fall 2015 – spring 2017), experimental plots were located in NE 20-28-07 W3. In year 2 (fall 2016 – spring 2018), the experimental plots were located adjacent to the year one plots in SE 20-28-07 WE.

Figure S2. Schematic illustrating how N application years (N-years) for spring and fall treatments were accounted for over the duration of the experiment

Figure S3. Ammonium-N (left two panels) and nitrate-N (right two panels) in the surface (0-15 cm) soil measured two weeks after the spring 2016 fertilizer application (4 May 2016) and 2-, 4-, and 6-weeks after seeding in spring 2016. Seeding date (4 May 2016) is represented by the black vertical arrow. Soils were collected on and off band. The bars represent the mean (n = 4).

Figure S4. Ammonium-N (left two panels) and nitrate-N (right two panels) in the surface (0-15 cm) soil measured two weeks after the fall 2016 fertilizer application (26 Oct 2016), 2-weeks prior to seeding, and 2-, 4-, and 6-weeks after seeding in spring 2017. Seeding date (12 May 2017) is represented by the black vertical arrow. Soils were collected on and off band. The bars represent the mean (n = 4).
